# Sexual Orientation Identity Development Milestones Among Lesbian, Gay, Bisexual, and Queer People: A Systematic Review and Meta-Analysis

**DOI:** 10.3389/fpsyg.2021.753954

**Published:** 2021-10-21

**Authors:** William J. Hall, Hayden C. Dawes, Nina Plocek

**Affiliations:** School of Social Work, University of North Carolina, Chapel Hill, NC, United States

**Keywords:** sexual orientation, identity development, milestones, gay, lesbian, bisexual, queer, sexual minority

## Abstract

This paper is a systematic review and meta-analysis on sexual orientation identity development milestones among people who are lesbian, gay, bisexual, or another sexual minority identity (LGB+). Common milestones measured in the 30 studies reviewed were becoming aware of queer attractions, questioning one’s sexual orientation, self-identifying as LGB+, coming out to others, engaging in sexual activity, and initiating a romantic relationship. Milestones occurred in different sequences, although attraction was almost always first, often followed by self-identification and/or sexual activity; coming out and initiating a romantic relationship often followed these milestones. Meta-analysis results showed that the mean effect sizes and 95% confidence intervals varied by milestone: attraction [*M*_age_=12.7 (10.1, 15.3)], questioning one’s orientation [*M*_age_=13.2 [12.8, 13.6]), self-identifying [*M*_age_=17.8 (11.6, 24.0)], sexual activity [*M*_age_=18.1 (17.6, 18.6)], coming out [*M*_age_=19.6 (17.2, 22.0)], and romantic relationship [*M*_age_=20.9 (13.2, 28.6)]. Nonetheless, results also showed substantial heterogeneity in the mean effect sizes. Additional meta-analyses showed that milestone timing varied by sex, sexual orientation, race/ethnicity, and birth cohort. Although patterns were found in LGB+ identity development, there was considerable diversity in milestone trajectories.

## Introduction

*Sexual orientation identity development* refers to changes, processes, and experiences over time that can involve awareness, exploration, appraisal, commitment, integration, and communication concerning a person’s identity as a sexual being, which is based on their patterns of sexual attractions and behaviors. Although commonalities exist in sexual orientation identity development, there are diverse trajectories across individuals and groups. For example, people who identify as lesbian, gay, bisexual, or another sexual minority identity (e.g., queer and pansexual; LGB+) navigate different tasks and milestones related to their stigmatized minority sexual orientation. *Sexual orientation* is a multidimensional construct, referring to an individual’s positioning on key dimensions of sexuality: sexual attraction, sexual behavior, romantic orientation, and sexual orientation identity (Mustanski et al., 2014; Hall, 2019). Historically, scholarship on LGB+ identity development was rooted in heterosexist notions and hindered by the limited number of completed studies; however, research in this area has continued to emerge and evolve. This article is a systematic review and meta-analysis of the recent empirical literature on sexual orientation identity development milestones among LGB+ people.

### Historical Overview of Scholarship on LGB+ Identity Development

Scholarship regarding the sexual orientation identity development of LGB+ people has evolved over the past 100years. For most of the 20th century, queer desires, behaviors, and identities that exist outside the norm of heterosexuality have been generally viewed as deviant and abnormal. According to Sigmund Freud’s theory of psychosexual development, humans were innately bisexual and became heterosexual or homosexual based on childhood experiences with parents (Freud, 1905). Freud theorized that homosexuality was a result of problems that arise during psychosexual development, such as boys becoming overly attached to and identifying with their mother instead of their father, feeling intense castration anxiety that leads boys to reject women because they are “castrated,” and narcissistic self-obsession that leads boys to choose an object of attraction that resembles themselves (Lewes, 1988). In later writings, Freud (1935) asserted that homosexuality was not a “vice,” “degradation,” or “illness.” Rather, he seemed to view homosexuality as an atypical variation in sexuality due to unresolved intrapsychic conflicts during childhood psychosexual development.

Psychoanalysts who followed Freud, including Sandor Rado, Irving Bieber, and Charles Socarides, took pathological views regarding homosexuality and asserted that homosexuality could be cured through psychoanalysis (Drescher, 2015). Rado (1940, 1949) rejected Freud’s assumption of innate bisexuality and contended that heterosexuality was the only natural and normal form of sexuality. Bieber (1962, 1967, 1969) claimed that male homosexuality was caused by boys having a possessive and overly involved mother, as well as a hostile or distant father; these dynamics led boys to bond with their mother and prevented them from developing their masculinity, which led him to effeminate homosexuality. For female homosexuality, Bieber (1967, 1969) claimed it was caused by various parent-child relationship dynamics, such as mothers being overly rejecting and critical of their daughters, showing little warmth and affection; this, combined with “defeminizing” behaviors, such as not dressing their daughter in pretty clothes and not teaching her cooking and housekeeping skills, contributed to homosexuality. Bieber (1962) also claimed that 27% of the patients in his clinical study were cured of their homosexuality. Socarides (1968) also maintained that homosexuality was an “illness” and “perversion” (p. 1) caused by problematic relationships between parents and their children. Socarides (1978) found a 44% success rate in curing patients of their homosexuality. Socarides also helped found the National Association of Research and Therapy of Homosexuality, which stated that “homosexuality is a treatable developmental disorder” (as cited in Mondimore, 1996, p. 224). These pathological views of homosexuality supplanted Freud’s theories and dominated psychology from the 1940s to the 1970s. Homosexuality was classified as an illness in 1952 with the initial publication of the Diagnostic and Statistical Manual of Mental Disorders (DSM).

Countering the psychopathology perspective was the work of Alfred Kinsey, which forwarded that homosexuality was a normal variation in human sexuality. Unlike prior psychoanalytic studies, Kinsey and his colleagues used non-psychiatric samples and found that homosexuality was not uncommon among men and women in the United States (Kinsey et al., 1948, 1953). Likewise, the work of Evelyn Hooker was also significant in countering the abnormality narrative of homosexuality. Hooker (1957) gathered psychological test results from heterosexual and gay men living in the community and then asked psychologists to appraise their psychological adjustment without knowing the participants’ sexual orientations. The psychologists classified the heterosexual and gay participants into equal levels of mental adjustment and they could not distinguish which participants were gay or heterosexual based on the test results. Hooker concluded that homosexuality was not inherently psychopathological. In 1973, members of the American Psychiatric Association voted on the removal of homosexuality from the DSM, which succeeded by a narrow majority of 58% (Drescher, 2015).

Sexual minority identity research during the 1970s, 1980s, and 1990s primarily focused on stage models to capture what was thought by many scholars to be a linear and universal progression in identity development (e.g., Cass, 1979, 1984, 1996). The Cass model has been the most widely recognized and cited of the stage models. The first stage of the Cass model involves initial awareness of feeling different in terms of sexual and/or romantic feelings or attractions that are not exclusively heterosexual. Next, the individual goes back and forth between feeling that they may likely be LGB+ to denial of this potential reality. Later, the person accepts the likelihood of being LGB+ but only later fully accepts a LGB+ identity. Next, the individual begins coming out to others, becomes involved with the LGB+ community and its culture, becomes highly aware of heterosexism and its effects, and may feel anger toward heterosexist people and institutions. In the final stage, the individual realizes that being LGB+ is just one part of who they are and synthesizes their LGB+ identity into a holistic sense of self.

However, critiques of the stage models and limited empirical validation of them (Kenneady and Oswalt, 2014; Savin-Williams and Cohen, 2015) led researchers to study the milestones that people experience as they develop a LGB+ identity. *Milestones* are events that mark significant points in human development in terms of life changes or achievements. Significant identity-related milestones for LGB+ people include one’s first awareness of queer or non-heterosexual desires, self-identifying as LGB+, and initially coming out as LGB+ to friends and family. Unlike the stage models, milestone-focused research did not presume a singular or ideal pathway of LGB+ identity development, but rather has attempted to examine patterns and variation in sexuality trajectories among LGB+ people, as well as to understand factors that shape the timing and sequence of milestones (e.g., Rosario et al., 1996; Savin-Williams and Diamond, 2000; Floyd and Stein, 2002; Maguen et al., 2002; Calzo et al., 2011). Milestone research has been conducted during the 1990s, 2000s, and 2010s.

### Implications of LGB+ Identity for Health and Psychosocial Functioning

In recent decades, scholars have also investigated disparities in mental and behavioral health problems facing LGB+ people. A growing body of evidence has documented many sexual orientation disparities where LGB+ people have significantly higher rates of psychological disorders and behavioral health problems compared to their heterosexual counterparts. Noteworthy disparities facing LGB+ people include depression, general anxiety, substance use/abuse (e.g., tobacco, alcohol, marijuana, and illicit drugs) and substance use disorders, suicidal ideation and suicidal behavior, eating disorders, body image and weight-related problems, and risky sexual behavior and sexually transmitted infections (e.g., Balsam et al., 2005; Marshal et al., 2008, 2011; Friedman et al., 2011; Everett, 2013; Fredriksen-Goldsen et al., 2013; Operario et al., 2015; Strutz et al., 2015; Choi and Meyer, 2016; Gonzales and Henning-Smith, 2017; Ross et al., 2018; Schuler et al., 2018; Miller and Luk, 2019; Rice et al., 2019; Kamody et al., 2020; Raifman et al., 2020). Making these disparities more alarming is evidence that they are present across the life span among LGB+ adolescents, adults, and older adults. These disparities have multiple causes, including negative experiences during LGB+ identity development. Research shows that failing to positively integrate one’s LGB+ identity into one’s overall identity predicts depression, anxiety, and low self-esteem (Rosario et al., 2011). Negative reactions from parents and friends after initially coming out as LGB+ are associated with low self-esteem, depression, suicidal ideation, and suicide attempts (Rosario et al., 2011; Baams et al., 2015; Ryan et al., 2015; Puckett et al., 2017).

Alternatively, many LGB+ people have positive identity development experiences that can contribute to psychological wellbeing and adaptive social functioning. The emerging scholarship on positive LGB+ identity (e.g., Baiocco et al., 2018; Riggle et al., 2014) indicates it is a psychosocial construct with multiple dimensions, such as enhanced self-awareness and personal insight, intrapersonal and interpersonal authenticity, freedom from gender roles, awareness of oppression and commitment to social justice, and connection with the queer community. Research shows that a positive LGB+ identity is inversely associated with depressive symptoms and positively associated with emotional self-awareness, self-compassion, emotional intimacy, social wellbeing, psychological wellbeing, and life satisfaction (Kertzner et al., 2009; Riggle et al., 2014; Riggle et al., 2017; Rostosky et al., 2018; Petrocchi et al., 2020). To reduce disparities and promote healthy and adaptive development among LGB+ people, we must understand the sexual orientation identity development process, which is a central aspect in the lives of LGB+ people.

### Purpose of This Review

Although several narrative literature reviews on this topic have been published (Reynolds and Hanjorgiris, 2000; Savin-Williams, 2011, 2019; Morgan, 2012; Mustanski et al., 2014; Savin-Williams and Cohen, 2015), they tend to focus on identity development for adolescents and emerging adults, and these reviews were not systematic reviews. Systematic reviews answer specific research questions on a topic using systematic, transparent, and replicable strategies to minimize bias and error. Systematic reviews are useful in understanding the state of the science in an area by summarizing what is known and moving science forward by providing directions for future research to improve upon limitations in prior studies and to address gaps identified in the literature. The purpose of this review was to systematically review the methodological characteristics and substantive findings of studies examining sexual orientation identity development milestones among LGB+ people. The following research questions drove this review: (1) What are the primary sexual orientation identity development milestones for LGB+ people? (2) At what ages do these milestones occur? (3) In what sequences to these milestones occur? (4) Does the chronology of milestones vary by sex, sexual orientation, race/ethnicity, or birth cohort? These four demographics were chosen because they are important biopsychosocial variables by which the timing of milestones may vary, and preliminary review of the literature indicated that there would be a sufficient number of studies to answer this research question, whereas there would be insufficient studies for other demographics (e.g., socioeconomic status, religious orientation, and ability/disability status).

## Materials and Methods

In preparing this review, the authors adhered to the Preferred Reporting Items for Systematic Reviews and Meta-Analyses criteria (Moher et al., 2015). Before undertaking the search for relevant studies, the authors developed a protocol for bibliographic database searches, study inclusion and exclusion criteria, and a data extraction tool. This review was registered with PROSPERO, an international database of systematic reviews regarding health and social wellbeing.

### Inclusion Criteria

Studies were included in the review if they met the following criteria: (1) collected data from lesbian, gay, bisexual, and/or queer people about the timing of their identity development milestones; (2) collected data in the United States; (3) were written in English; and (4) were published or completed on or after January 1, 1990. The time period selected allowed for a contemporary review of the empirical literature completed since the shift in focus toward understanding sexuality milestones. Studies conducted outside the United States were excluded because different countries typically have different social constructions of sexual orientation, cultural values about sexuality, and institutional policies and practices about sexual orientation (Human Rights Watch, 2009; Pew Research Center, 2013b).

### Search Procedure

A behavioral and social sciences librarian was consulted to assist with developing a search string and identifying relevant computerized bibliographic databases in which to search. The following search string was used to search the *PsycINFO* and *Sociological Abstracts* databases for studies published between January 1, 1990 and September 4, 2019 (i.e., the day the searches were performed): (identity OR milestone OR development) in Abstract AND (gay OR lesbian OR bisexual OR homosexual OR queer OR “sexual minority” OR “sexual minorities”) in Abstract AND (“sexual orientation” in Subjects for *PsycINFO*; sexuality in Subject Heading for *Sociological Abstracts*). The EBSCO platform was used for the *PsycINFO* searches and ProQuest was used for the *Sociological Abstracts* searches. The English language filter was also used in the searches. The *PsycINFO* and *Sociological Abstracts* databases include published empirical literature, as well as gray literature sources (e.g., unpublished dissertations, conference proceedings, and working papers). These more formal bibliographic database searches were supplemented with searches of Google Scholar, which also contains published and gray literature. The first 100 Google Scholar search results were examined.

### Study Screening

**Figure 1:**
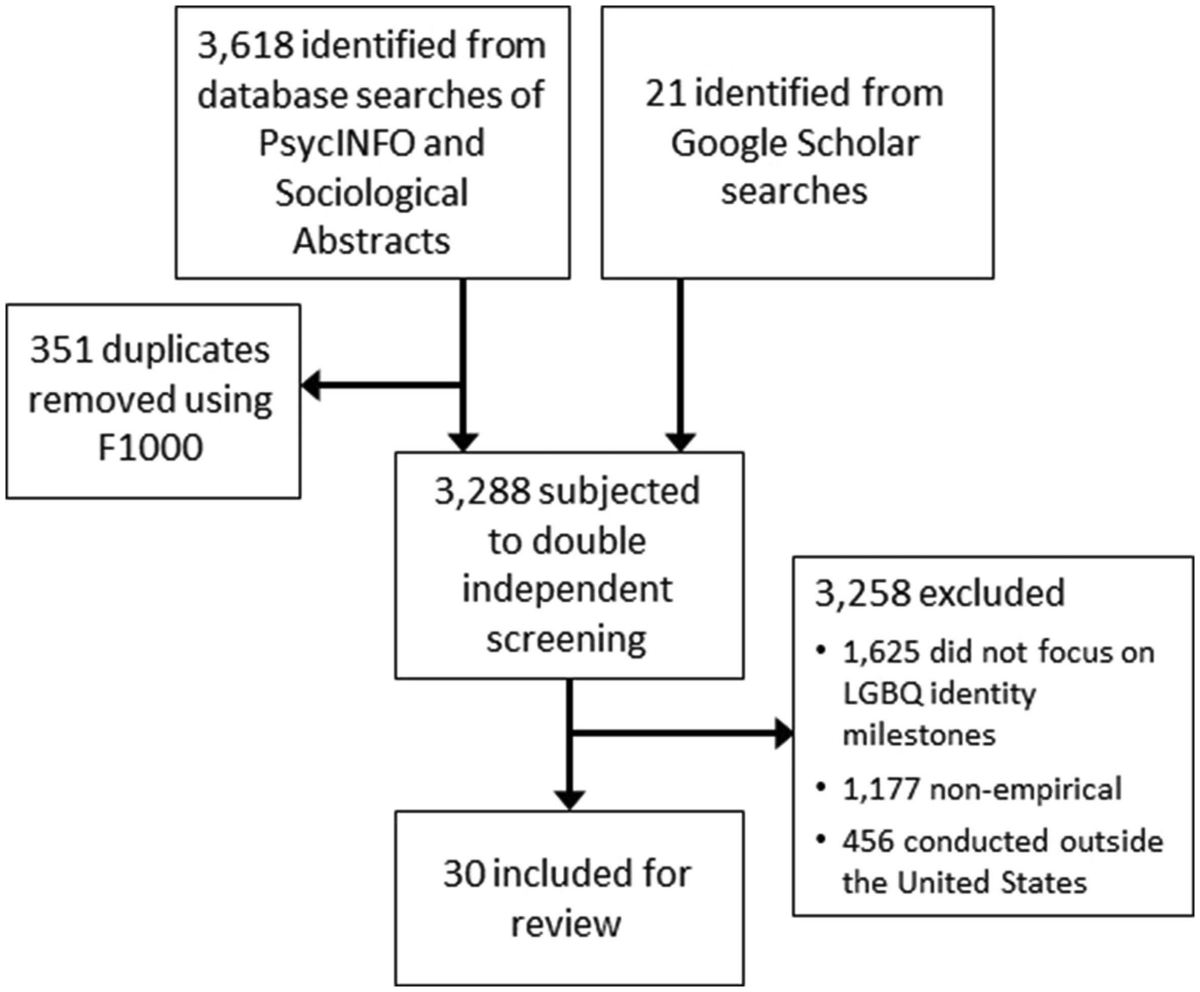
Flowchart Depicting the Identification, Screening and Inclusion or Exclusion of Studies.

After performing the bibliographic searches, 3,618 results were imported into the F1000 program to assist with organization and duplicate removal. Following duplicate removal, 3,267 studies remained. The first author and a trained research assistant independently screened each study to determine eligibility. A checklist of the inclusion and exclusion criteria was created prior to the search and was used for eligibility assessment. Most studies were included or excluded after reading the title and abstract; however, it was also necessary to examine the full-text documents of some studies to determine eligibility. To examine inter-rater agreement, the decisions of the two screeners were compared and Cohen’s kappa statistics were calculated with SPSS (version 21), which showed excellent agreement: kappa=0.96, *p*<0.001. There were only 14 disagreements between the screeners, which were resolved by the first author examining the full-text documents. After screening, 3,258 studies were excluded because they did not meet all of the inclusion criteria. More specifically, 1,625 studies were excluded because they did not focus on sexual orientation identity development milestones among LGB+ people, 1,177 studies were excluded because they were not empirical papers (e.g., theoretical or conceptual papers, book reviews, introductions to special issues, commentaries, and position statements), and 456 studies were excluded because they were conducted outside of the United States. After completing the search and screening processes, 30 studies were included for extraction and review (Figure 1).

### Data Extraction

A data extraction spreadsheet was developed to assist with identifying and collecting relevant information from the 30 included studies. Information extracted included the citation, study design, sampling strategy and location, sample size and characteristics, time of data collection, types of milestones measured, timing of milestones, sequences of milestones, and comparisons of milestone timing by group characteristics of interest (i.e., sex, race/ethnicity, sexual orientation, and birth cohort). The first author extracted this information, and then a trained research assistant compared the extraction spreadsheet completed by the first author with the full-text documents to check the accuracy of the extractions. There were only four points of disagreement between the extractor and checker, which were resolved together by examining the full-text documents and extractions simultaneously.

### Data Synthesis

#### Meta-Analysis Methods

Initial review of the included studies revealed that a quantitative synthesis of milestone timing in the form of a meta-analysis could be undertaken but would need to be conducted selectively because of the methodological heterogeneity of the studies. This heterogeneity included variation in the rigor of study designs and methods, which indicated limitations regarding internal and external validity for some studies. For example, most studies used non-probability sampling, only one-third of studies used national United States samples, and over one-quarter of studies had relatively small sample sizes (*N*<200). Therefore, instead of performing meta-analyses of milestone ages using data from all 30 studies, we used the best available evidence from a select group of studies. The best evidence was from two studies that used national probability sampling and had large samples (*N*>650; Herek et al., 2010; Pew Research Center, 2013a). Meta-analysis can be performed with data from two studies (Borenstein et al., 2009); however, these two studies did not measure all of the main milestones identified in the literature. Therefore, in these instances, we used data from studies from the next level of methodological rigor: those that used national non-probability sampling and relatively large sample sizes (*N*>500; Fox, 1993; D’Augelli, 2002; Morris et al., 2002; Drasin et al., 2008; Dunlap, 2016; Fredriksen-Goldsen et al., 2017; Katz-Wise et al., 2017a; Grov et al., 2018). Many of these studies also reported milestone ages by sex, sexual orientation, and birth cohort, but not by race/ethnicity. Therefore, meta-analyses for milestone ages by race/ethnicity used the handful of studies that reported these data (Dubé, 1997; Parks et al., 2004; Martos et al., 2015; Hoenig, 2016; Fredriksen-Goldsen et al., 2017), though most of them were not studies in the top or second tiers of methodological rigor. Meta-analyses by sex, sexual orientation, race/ethnicity, and birth cohort were performed on five of the primary milestones of interest because data for other milestones were not available.

Multiple meta-analysis models were run, one for each of the milestones of interest. All the analyses used mean age as the effect size metric. When effect size data needed for meta-analysis (i.e., standard error of the mean) were reported in other formats (i.e., standard deviation), the data were transformed using formulas recommended by Higgins et al. (2019). Meta-analyses were performed using the Meta-Essential program (version 1.5; Van Rhee et al., 2015; Suurmond et al., 2017). Random-effect models were used because we assumed that the true mean effects could vary across samples and studies. An inverse variance weighting method with an additive between-studies variance component based on the DerSimonian-Laird estimator was used (Van Rhee et al., 2015). The confidence intervals (CIs) for the mean effects were estimated using the weighted variance method for random-effect models (Sánchez-Meca and Marín-Martínez, 2008), and the CIs for the individual study effect sizes were calculated using the Student’s t-distribution. Three studies (Drasin et al., 2008; Herek et al., 2010; Grov et al., 2018) were missing data needed for the standard error (SE) of the mean. In these cases, mean imputation was used to replace the missing value with the mean of the SEs from the other studies for the same milestone. Outliers were identified if the CI of an individual study effect size did not overlap with the CI of the overall mean effect size (Harrer et al., 2019). Following recommendations from Lipsey and Wilson (2001), outliers were Winsorized by recoding each to the next eligible nearest neighbor.

#### Narrative Synthesis

We also used a narrative synthesis approach recommended by Petticrew and Roberts (2006) to combine findings across the 30 studies. Following this approach, findings from studies were grouped under the four research questions they corresponded to. Next, the findings were examined in each domain to identify themes, including dominant trends and patterns as well as variation in findings, such as results that deviated from dominant trends. Descriptive summaries of the evidence synthesis were then crafted for each research question. A narrative summary of the methodological characteristics of the 30 studies was also created to illustrate the strengths and limitations of the studies.

## Results

A total of 30 studies were included in this review (Supplementary Table 1). Given that multiple publications or research products may have derived from the same study, we use the term “study” to refer to the original research project where data were collected from a unique sample. For simplicity, in-text citations to the 30 studies will use the first citation listed in Supplementary Table 1 for multiple citations from the same study. Next, we will present a summary appraisal of the methodological characteristics of the 30 studies. Supplementary Table 1 displays an overview of the methodological characteristics of the studies. Then, we present the results of the evidence syntheses for each research question: (1) LGB+ identity development milestones measured; (2) the timing of these milestones; (3) the sequences of these milestones; and (4) comparisons of milestone timing by sex, race/ethnicity, sexual orientation, and birth cohort.

### Methodological Quality of Studies

#### Designs

In terms of study design, 20 studies were cross-sectional with four of those 20 using multiple cross-sectional samples, and 10 studies used longitudinal designs. However, the studies with longitudinal designs only measured milestones at one wave retrospectively. Therefore, no studies used prospective longitudinal methods to assess milestones. Almost all studies (*n*=28) used quantitative methods; only two studies used mixed methods. Four studies used some form of probability sampling with two of these studies using national probability sampling; 26 studies used some form of non-probability sampling (e.g., convenience sampling, purposive sampling, snowball sampling, and quota sampling). One-third of studies (*n*=10) used national United States samples, nine studies sampled participants from a single city or urban area (e.g., San Francisco Bay Area), five studies sampled participants from a single state, four studies sampled participants from multiple cities, and two studies sampled participants from one or two United States regions (e.g., the Midwest). Among the non-national studies, samples were primarily drawn from large metropolitan areas (i.e., Chicago, Los Angeles, New York City, and San Francisco). Few studies sampled participants from the Southeast, Southwest, and Mountain-Prairie regions.

#### Samples

Sample sizes ranged from 16 to 2,733 participants across studies (*M*=890, *SD*=811). The average age of participants in study samples varied from 17.0 to 61.5 (*M*=30.8, *SD*=11.1). Half of the samples (*n*=15) were relatively equal in terms of males and females, eight studies were exclusively or primarily male samples, and seven studies were exclusively or primarily female samples. Hardly any studies reported participants’ gender identities. In terms of sexual orientation, nine studies had samples that were exclusively or primarily (i.e., 82–100%) gay/lesbian-identified participants with the remaining participants identifying as bisexual; nine studies had samples of large majorities (i.e., 60–76%) of gay/lesbian participants with smaller representation of bisexual, queer, and other sexual minority identities; five studies had samples of relatively equal numbers of gay/lesbian and bisexual participants; three studies included participants with substantial representation of gay/lesbian, bisexual, and other sexual orientation identities; one study consisted of only bisexual participants; and three studies did not provide breakdowns for sexual orientation identities. Only 10 studies reported proportions of sexual orientation identities other than gay, lesbian, or bisexual, such as queer, pansexual, and mostly gay/lesbian. In terms of the racial/ethnic breakdowns of samples, about two-thirds of studies (*n*=19) consisted of samples that were largely White (i.e., 65–91% White participants). Less than half of the studies included samples with at least 10% Black/African Americans or at least 10% Hispanic/Latinx Americans. Only 17 studies reported the proportion of Asian American participants, and nine of these studies included at least 5% Asian Americans. Nine studies reported the proportion of Native American participants, which ranged from 1 to 3%. Very few studies reported participants who identified as multiracial/multiethnic. In addition, few studies reported other participant demographics, including gender identity, socioeconomic status, national origin, religious background, and ability/disability status. The years data were collected during studies ranged from 1987 to 2015 (*M*=2001, *SD*=8.0). In terms of birth cohorts included in samples, 20 studies included participants from Generation X (born 1965–1980), 17 studies included participants from the Baby Boom Generation (born 1946–1964), 15 studies included participants from the Millennial Generation or Generation Y (born 1981–1996), 12 studies included participants from the Silent Generation (born 1928–1945), and only one study included participants from Generation Z (born 1997–2012). These cohort names and birth years are based on definitions by the Pew Research Center (2015, 2019b).

### Milestones Measured

Studies varied regarding which milestones were assessed. Four milestones were measured in the large majority studies: self-identifying as LGB+ (*n*=28), coming out to others (*n*=24), engaging in same-sex sexual activity (*n*=23), and becoming aware of queer attractions or desires (*n*=22). Other milestones assessed in some studies included questioning one’s sexual orientation (*n*=9), having a romantic relationship (*n*=8), experiencing queer fantasies (*n*=4), and experiencing feelings of differentness (*n*=2). Although most studies assessed coming out generally (e.g., “When did you first tell someone that you are LGB?”), other studies assessed coming out in specific social contexts, including parents (*n*=8), family members besides parents (*n*=6), family in general (*n*=2), friends (*n*=3), and individuals who are LGBTQ (lesbian, gay, bisexual, transgender, or queer) (*n*=1).

### Timing of Milestones

Supplementary Table 2 shows the timing of the eight most commonly measured milestones across the 30 studies. The meta-analysis results are shown in the last row. These results are based on the best available evidence from 10 studies representing 13,299 total participants from probability and non-probability national samples. Although there is diversity in the samples in terms of age, birth cohort, sex, sexual orientation, and race/ethnicity; males, gay/lesbian people, and White people were slightly overrepresented in the non-probability samples. Only one study included participants from Generation Z, which is understandable because it is the most recent birth cohort. The meta-analysis results show that on average attraction and questioning one’s orientation occurred around age 13, which was based on data from eight studies in the second tier of methodological rigor for attraction and one study in the top tier of methodological rigor for questioning. Self-identifying as LGB+ and engaging in sexual activity occurred around age 18, which were based on two studies in the top tier of evidence for self-identifying and eight studies in the second tier of methodological rigor for sexual activity. Having a romantic relationship occurred around age 21, which was based on eight studies in the second tier of evidence. Coming out as LGB+ to others in general occurred around age 19 to 20, which was based on two studies in the top tier of evidence. Coming out to parents and other family members occurred a bit later, around age 23, which is based on eight studies in the second tier of methodological rigor. All of the results showed substantial heterogeneity in the mean effect sizes, which is expected because the ages individuals reach milestones may likely vary by sex, gender, sexual orientation, birth cohort, race/ethnicity, and geographic location. The CIs indicate the ranges where the true mean ages would fall in 95% of comparable studies. For example, the average age that individuals self-identify as LGB+ was 17.8 (Supplementary Table 2). The true mean age likely falls in the range of 11.6 to 24.0 in 95% of studies with methods similar to Herek et al. (2010) and Pew Research Center (2013a), which were the studies used in that meta-analysis.

### Sequences of Milestones

**Figure 2:**
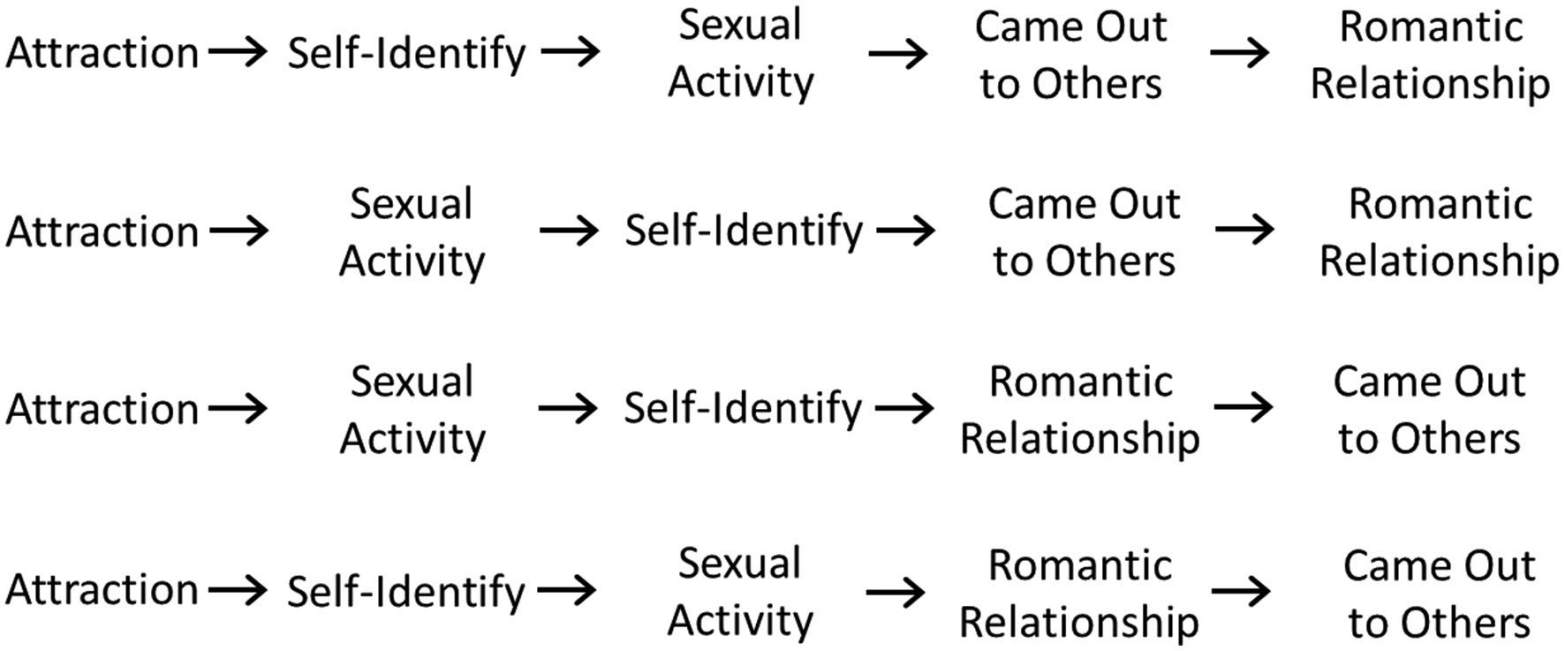
Common Sequences of LGB+ Identity Development Milestones.

Based on the last row of Supplementary Table 2, meta-analysis results show the most common milestone sequence is (1) becoming aware of queer attractions, (2) questioning one’s orientation, (3) self-identifying as LGB+, (4) engaging in sexual activity, (5) coming out to others, and (6) having a romantic relationship. However, the results from the studies showed that LGB+ people experienced the milestones in many different sequences. Figure 2 shows the four most common sequences for the five most commonly measured milestones. Attraction was almost universally the first milestone experienced. Only one study found that a small group of participants (5% of the sample) had engaged in sexual activity prior to becoming aware of same-sex attractions (Maguen et al., 2002). Generally, the second and third milestones were either self-identifying as LGB+ or engaging in sexual activity. The latter milestones were typically coming out to others and one’s first romantic relationship. Findings also showed that for some individuals, certain milestones did not occur consecutively but rather concurrently. For example, for some people, awareness of their attractions occurred at the same age they engaged in sexual activity or came out to others. Similarly, at the age individuals self-identified as LGB+, some were also engaging in sexual activity, coming out, or initiating romantic relationships. And while some individuals were coming out as LGB+, they were also engaging in sexual activity or having a romantic relationship.

### Milestone Chronology by Sex, Race/Ethnicity, Sexual Orientation, and Birth Cohort

#### Milestone Comparisons by Sex

Among the studies that compared the timing of milestones by sex (male vs. female) in their analyses, results showed statistically significant differences for some milestones but not others. Eight studies found that males experienced awareness of attractions earlier than females (Fox, 1993; Savin-Williams and Diamond, 2000; D’Augelli, 2002; Floyd and Bakeman, 2006; D’Augelli et al., 2008; Hoenig, 2016; Katz-Wise et al., 2017a), and three studies found no significant differences (Herdt and Boxer, 1993; Rosario et al., 1996; Martos et al., 2015). Another study found that females experienced attraction later than males, except among Millennials in which there were no significant differences (Dunlap, 2016). Regarding self-identification as LGB+, nine studies showed that males self-identified earlier than females (Fox, 1993; Rosario et al., 1996; Savin-Williams and Diamond, 2000; D’Augelli, 2002; Floyd and Bakeman, 2006; Grov et al., 2006; Herek et al., 2010; Martos et al., 2015; Katz-Wise et al., 2017a), and three studies found no significant differences (Herdt and Boxer, 1993; Hoenig, 2016). Two other studies found that males generally self-identified earlier than females, but this also depended on their cohort, with no significant differences between males and females among Millennials (Dunlap, 2016) and among individuals whose sexual identity development started later in life (Calzo et al., 2011). For first sexual activity, seven studies found that males initiated sexual activity before females (Fox, 1993; Herdt and Boxer, 1993; Savin-Williams and Diamond, 2000; Floyd and Bakeman, 2006; Grov et al., 2006; Calzo et al., 2011; Katz-Wise et al., 2017a), and two studies found no significant difference (Rosario et al., 1996; Hoenig, 2016). Another study found that females had first sexual activity later than males, except among Millennials in which there were no significant differences (Dunlap, 2016). Two studies found that males had their first romantic relationship earlier than females (Fox, 1993; Martos et al., 2015), one study found that females had a romantic relationship before males (D’Augelli, 2002), and a fourth study found no significant differences (Dunlap, 2016). Regarding coming out to others, eight studies found no significant differences by sex for the disclosure milestone (Fox, 1993; Savin-Williams and Diamond, 2000; D’Augelli, 2002; Floyd and Bakeman, 2006; Grov et al., 2006; D’Augelli et al., 2008; Martos et al., 2015; Hoenig, 2016). Another study found no significant difference in coming out between males and females, except in the Silent Generation, when males came out earlier than females (Dunlap, 2016). And, another study found that among individuals whose sexual identity development started later in life, females came out earlier than males (Calzo et al., 2011). A final study showed that males came out earlier than females (Herek et al., 2010).

Supplementary Table 3 shows the results of meta-analyses of milestone ages for females and males based on data from six studies that provided milestone ages by sex; five of these studies are in the second tier of methodological rigor and one study is in the top tier (Fox, 1993; D’Augelli, 2002; Herek et al., 2010; Dunlap, 2016; Fredriksen-Goldsen et al., 2017; Katz-Wise et al., 2017a). As Supplementary Table 3 shows, on average, males experienced the attraction, self-identification, and sexual activity milestones earlier than females. However, males and females came out and had their first romantic relationship at about the same ages, though females were slightly earlier. The order of milestones was similar for males and females beginning with attraction, followed by self-identification, sexual activity, romantic relationship, and disclosure. Finally, despite starting later than males, females achieved all five of the main milestones in a shorter amount of time (15.3 to 21.9=6.6years), compared to males (12.9 to 22.3=9.4years).

#### Milestone Comparisons by Race/Ethnicity

Among the studies that compared the timing of milestones between racial/ethnic groups in their analyses, three studies found no statistically significant differences between groups for any milestone (Rosario et al., 1996; Herek et al., 2010; Martos et al., 2015). Nevertheless, four other studies found some significant differences for certain milestones but not others. In a study of male youth, Hispanic/Latino youth reported awareness of attractions earlier than Black and White youth, and Asian youth reported first sexual activity later than White, Black, and Hispanic/Latino youth (Dubé and Savin-Williams, 1999). Similarly, a study of young adults found that Hispanic/Latina women reported initial sexual activity earlier than White women, and Black men reported initial sexual activity earlier than Asian, White, and other racial/ethnic men (Grov et al., 2006). Another study of youth found no significant differences in milestone timing except that Black youth self-identified and came out later than Latinx youth (Hoenig, 2016). In a study of gay/lesbian women, there were no significant differences between Black and Hispanic/Latina women, but compared to women of color, White women were significantly later in questioning their orientation, self-identifying as gay/lesbian, coming out, and having a same-sex romantic relationship (Parks et al., 2004).

Supplementary Table 3 shows the results of meta-analyses of milestone ages by race/ethnicity based on data from five studies that provided age breakdowns by race/ethnicity; four of these studies are in the third tier of methodological rigor and one study is in the second tier (Dubé and Savin-Williams, 1999; Parks et al., 2004; Martos et al., 2015; Hoenig, 2016; Fredriksen-Goldsen et al., 2017). Supplementary Table 3 shows that Hispanic/Latinx individuals experienced attractions earliest, followed by Asian and White individuals, with Black/African American individuals reporting attraction latest. Racial/ethnic differences for ages when individuals self-identified as LGB+ are limited, with Hispanic/Latinx and Asian people self-identifying slightly earlier than Black/African American and White people. Regarding initial sexual activity, Black/African American and Hispanic/Latinx people engaged in sexual activity slightly earlier, followed by White and then Asian individuals. Similarly, Hispanic/Latinx and Black/African American individuals reported having romantic relationships and coming out slightly earlier than Asian and White individuals. Regarding milestone sequences, all groups followed a sequence of attraction, sexual activity, self-identify, romantic relationship, and coming out. Despite starting latest, Black/African American people achieved all five of the milestones in the shortest amount of time (12.3 to 20.5=8.2years), followed by Hispanic/Latinx people (10.6 to 19.8=9.2), Asians (11.3 to 21.1=9.8), and White people (11.4 to 21.2=9.8).

#### Milestone Comparisons by Sexual Orientation

Among the seven studies that compared the timing of milestones between gay/lesbian and bisexual people in their analyses, most studies found that gay/lesbian people reached the milestones of attraction and self-identifying significantly earlier than bisexual people (Diamond, 1998; Maguen et al., 2002; Herek et al., 2010; Calzo et al., 2011; Martos et al., 2015; Hoenig, 2016; Katz-Wise et al., 2017a). Only one study examined first romantic relationship and found no significant sexual orientation differences (Martos et al., 2015). Few studies examined comparisons for sexual activity and coming out, and these results were more mixed and nuanced. For example, in two studies, gender played an interactive role. In one study, attraction was experienced earlier for gay men than bisexual men, with no differences between gay/lesbian women and bisexual women (Katz-Wise et al., 2017a). Similarly, another study found that sexual activity was earlier for gay men than bisexual men, with no differences among women; coming out was earlier for gay/lesbian women than bisexual women, with no difference among men (Maguen et al., 2002).

Supplementary Table 3 shows the results of meta-analyses of milestone ages by sexual orientation (bisexual and gay/lesbian) based on data from four studies; two of these studies are in the top tier of methodological rigor and two are in the second tier (Herek et al., 2010; Pew Research Center, 2013a; Fredriksen-Goldsen et al., 2017; Katz-Wise et al., 2017a); however, none of these studies examined the relationship milestone. Gay/lesbian people experienced the milestones earlier than bisexual people—about 1 to 2years for attraction, self-identifying, and sexual activity. However, coming out to others occurred at about the same age for both groups. In terms of milestone sequencing, bisexual and gay/lesbian people experienced attraction, followed by sexual activity, self-identification, and coming out. Despite starting later, bisexual people completed the milestones in a shorter amount of time (16.9 to 21.6=4.7years) compared to gay/lesbian people (15.1 to 21.3=6.2).

#### Milestone Comparisons by Birth Cohort

Among the studies that compared the timing of milestones between birth cohorts, all found significant differences between cohorts for the milestones of self-identifying, coming out, and romantic relationship whereby older cohorts experienced milestones later in life compared to younger cohorts (Fox, 1993; Floyd and Stein, 2002; Floyd and Bakeman, 2006; Grov et al., 2006, 2018; Parks and Hughes, 2007; Drasin et al., 2008; Herek et al., 2010; Calzo et al., 2011; Pew Research Center, 2013a; Martos et al., 2015; Dunlap, 2016; Fredriksen-Goldsen et al., 2017). However, findings were less consistent for the milestones of attraction and sexual activity. Four studies did not find significant differences in age of first attraction, but this finding only applied to males (Drasin et al., 2008; Martos et al., 2015; Dunlap, 2016; Grov et al., 2018). Similarly, three studies did not find substantial differences between cohorts in age of initial sexual activity among males (Grov et al., 2006; Drasin et al., 2008; Dunlap, 2016). In addition, although studies found significant cohort differences in age of self-identifying, these differences were smaller in several studies compared to the milestones of first disclosure and romantic relationship (Drasin et al., 2008; Martos et al., 2015; Fredriksen-Goldsen et al., 2017).

Supplementary Table 3 shows the results of meta-analyses of milestone ages by birth cohort based on data from five studies; four of these studies are in the second tier of methodological rigor and one study is in the top tier (Fox, 1993; Pew Research Center, 2013a; Dunlap, 2016; Grov et al., 2018). The timing of milestones was earliest for Millennials, followed by Generation X, Baby Boomers, and the Silent Generation. Millennials also achieved all five of the milestones in the shortest amount of time (12.0 to 17.9=5.9years), followed by Generation X (12.7 to 20.1=7.4years), Baby Boomers (13.1 to 24.3=11.2years), and the Silent Generation (15.0 to 29.2=14.2years). Millennials also differed from the other birth cohorts in milestone sequence with sexual activity following self-identifying as LGB+, whereas sexual activity preceded self-identifying as LGB+ for Generation X and Baby Boomers, and the Silent Generation self-identified as LGB+ and engaged at sexual activity at the same age. The attraction milestone showed limited differences between Millennials, Generation X, and Baby Boomers occurring at age 12 to 13, whereas the Silent Generation experienced attraction at 15. The romantic relationship milestone shows the most differences between birth cohorts.

## Discussion

The purpose of this article was to systematically review studies examining sexual orientation identity development milestones among LGB+ people. The findings will be discussed according to the research questions that drove the review.

### Milestones Measured

Four milestones were measured in about 75% of studies (i.e., self-identifying, coming out generally, sexual activity, and awareness of attractions), another four milestones were measured in about 20–30% of studies (i.e., questioning one’s sexual orientation, romantic relationship, and coming out to parents and family members), and another set of four milestones were measured in only a handful of studies (i.e., sexual fantasies, feeling different, and coming out to friends and LGBTQ people). This indicates that researchers view certain milestones as more significant in the development of a LGB+ identity. Initial awareness of queer attractions or desires is typically experienced first, making it an important initial marker in sexuality development. However, there are multiple related yet different experiences that can be considered attraction/desire (Foster, 2009; Diamond, 2010). One component, *proceptivity*, encompasses urges to seek sexual activity. Another is *arousability*, the capacity to become interested in sexual activity after encountering erotic stimuli. Attraction can also be physical or romantic in nature, with *romantic attraction* involving desire for emotional intimacy, dating, or a romantic relationship. None of the studies reviewed operationalized attraction with these three dimensions.

Sexual activity was another milestone that was frequently measured with measurement limitations. First sexual activity is an important developmental event that has implications for health and psychosocial wellbeing. The large majority of studies assessed sexual activity with a general question (e.g., “How old were you when you first had a sexual encounter with someone of the same sex?”) rather than a series of items about various sexual acts (e.g., penile-vaginal intercourse, penile-anal intercourse, oral sex, and manual stimulation). Although a single general question to measure sexual activity is advantageous in terms of feasibility and respondent burden, individuals’ understandings of what acts constitute sex varies; for example, some individuals do not consider oral or manual stimulation of the genitals to be sex (Bogart et al., 2000; Peterson and Muehlenhard, 2007; Sanders et al., 2010). Use of a single general question about sexual activity allows for openness to interpretation by respondents who have various notions about what forms of contact count as sex.

Choosing an identity label that best fits one’s experiences and applying that label to one’s self is a self-defining moment and perhaps the most fundamental of the milestones in sexual orientation identity development. It should be noted that this milestone can occur at multiple points in development. Research shows that a substantial minority of people change their identity label over time (Ott et al., 2011; Mock and Eibach, 2012; Savin-Williams et al., 2012; Fredriksen-Goldsen et al., 2017). For example, some people who initially identify as bisexual later identify as gay/lesbian, mostly heterosexual, or completely heterosexual. Those who report being unsure of their identity often later identified as bisexual, gay/lesbian, or heterosexual. And some people who initially identified as mostly heterosexual later identified as bisexual or completely heterosexual. These findings show that there is fluidity in sexual orientation, though most individuals report stability in their self-identification. Researchers have also found new identity labels being used and variation in the meanings of identities, with the same identity label having different meanings from person to person (Rust, 2000; Walton et al., 2016; Galupo et al., 2017; Hall, 2019; Watson et al., 2020). This is unsurprising because sexual orientation identities are socially constructed.

The first time a person discloses their LGB+ identity to someone else is another important milestone given societal heteronormativity where people are presumed to be heterosexual unless otherwise stated. Coming out to others is not a one-time event, but rather a multitude of events occurring over the life course with various people (e.g., family members, friends, classmates, and co-workers) and settings (e.g., home, neighborhood, school, workplace, place of worship, and social media). Nonetheless, the first time someone discloses their LGB+ identity to a friend or family member may be particularly vulnerable for the person coming out, and the nature of reactions of others can significantly influence mental health and interpersonal relationships in positive and negative ways (Beals and Peplau, 2006; Rosario et al., 2009; Rothman et al., 2012; D’Amico et al., 2015; Ryan et al., 2015). The emotional and behavioral reactions of others to disclosure can vary considerably from affirmation to rejection.

Certain milestones (i.e., feeling different and questioning one’s sexual orientation) may have been less frequently measured because they are more ambiguous in nature and less of a distinct, salient event. It should also be noted that despite the pervasiveness of heteronormativity, not all LGB+ people may experience feelings of differentness and question their orientation. Traditionally, children have been raised under the presumption of heterosexuality because a heterosexual identity is the norm and ideal in United States society. However, recently emerged parenting practices challenge presumptions of heteronormativity among children and adolescents by forwarding sexual diversity as the social norm with multiple valued sexual identities, including LGB+ identities (Helsel and Harris-Smith, 2020; Planned Parenthood, 2020). Young people growing up with this socialization with an emerging LGB+ identity may not feel confused, uncertain, or out-of-place when LGB+ feelings arise.

### Timing of Milestones

The meta-analysis results show that, on average, attraction and questioning one’s orientation occurred during early adolescence, whereas self-identifying as LGB+, sexual activity, and initially coming out occurred during late adolescence; coming out to parents and other family members and having a romantic relationship occurred during emerging adulthood. The general timing of these milestones occurring during adolescence and emerging adulthood is expected because adolescence is a time of self-discovery and self-definition, as well as a blossoming in sexual feelings and experiences following puberty (Hall and Rounds, 2013). Nonetheless, the broad CIs and SDs for mean milestone ages (Supplementary Tables 2 and 3) demonstrate that for some individuals, milestones were experienced as early as childhood or as late as middle adulthood.

When an individual reaches a milestone, the developmental context has significant implications for how they experience the milestone and its impact on their biopsychosocial trajectories (Hall et al., 2022). For example, a person who becomes aware of same-sex attractions and questions their orientation during early adolescence may have less self-knowledge and insight about sexuality or have access to fewer resources to help them process and make sense of their experience, compared to someone in late adolescence. And, for example, someone who comes out to their parents during early adolescence versus later, such as late adolescence or young adulthood, would likely have different experiences with varying developmental implications. One’s parents may be more accepting or rejecting of their child’s identity. Hostile parental reactions could have more negative effects on the child’s mental health, housing stability, and financial status if the child is 13years old compared to age 18 or 25, when children are generally more independent and psychologically autonomous from their parents. Conversely, coming out to parents at an earlier age with neutral or positive reactions may preclude stressors from remaining in the closet to one’s family until a later age (e.g., hiding one’s identity, feeling dishonest, and interpersonal distance) and be beneficial for the individual and the parent-child relationship. Stress related to hiding one’s LGB+ identity is associated with depressive symptoms (Hall, 2018).

Although some of the sexual orientation identity development milestones are unique to LGB+ people (e.g., coming out), others are experienced across the human condition regardless of sexual orientation (e.g., attraction). The average age of first sexual attraction found in our analysis for LGB+ people (i.e., 12.7years) corresponds with results from studies with the general population (Bancroft et al., 2003; Reynolds et al., 2003). The timing of attraction is presumably biologically based, with the onset of desire to seek sexual activity following gonadarche (i.e., pubertal changes involving the growth of the ovaries and testes and hormonal increases in estrogen and testosterone), which typically begins around age 11–12 (Wan et al., 2012; Chulani and Gordon, 2014; Diamond et al., 2015; Wohlfahrt-Veje et al., 2016). Nonetheless, the capacity for arousability can occur following adrenarche (i.e., pre-pubertal increases in adrenal androgen hormones), which begins during ages 5–8 (Rainey et al., 2002; Remer et al., 2005; Diamond et al., 2015).

Another common milestone is first sexual contact, which we found occurred on average at age 18.1. Studies with the general population have found that first sexual activity occurs around age 17 (Haydon et al., 2012; National Center for Health Statistics, 2017), which is slightly earlier. LGB+ individuals may initiate sexual activity slightly later than their heterosexual counterparts because they may be working through issues specific to their emerging LGB+ identity. For example, unlike heterosexual individuals, LGB+ people may need time to process their attractions considered abnormal or unacceptable by society, explore their sexuality, choose an identity label that fits their experiences, and confront sexual stigma. In addition, LGB+ sexual activity may be delayed because many young people have not received affirmative, accurate, or comprehensive education about LGB+ sexual activity. Indeed, researchers have found that sex education typically focuses on heterosexuality; ignores or stigmatizes LGB+ sexualities; and emphasizes abstinence, often abstinence until heterosexual marriage (McNeill, 2013; Estes, 2017; Hall et al., 2019).

Meta-analytic results of the age of one’s first LGB+ romantic relationship (i.e., 20.9) were significantly later than what studies have found for heterosexual individuals. Research with heterosexual samples shows that one’s first romantic relationship occurred around age 16.6–18 (Regan et al., 2004; Zimmer-Gembeck et al., 2004; Raffaelli, 2005; Smiler et al., 2011). The later onset of romantic relationship initiation for LGB+ people may be due to several factors. LGB+ individuals would likely not initiate a queer relationship until after they have self-identified as LGB+ and started coming out, which we found occurred at ages 17.8 and 19.6, respectively. Socio-historically, before the proliferation of LGBTQ community centers and organized groups as well as LGBTQ-specific online dating services and apps, older generations of LGB+ people often relied on LGBTQ bars and night clubs to meet prospective romantic partners, and these institutions had age restrictions of 18 or 21. Internalized stigma may also play a role whereby individuals with higher levels of internalized stigma may forgo LGB+ relationships because they have internalized societal views that queer relationships are immoral, abnormal, or unhealthy. Findings from one study showed that internalized stigma was associated with loneliness, lack of warmth and trust in interpersonal relationships, and inhibition in sexual desire (Frost and Meyer, 2009).

### Sequences of Milestones

The results demonstrate that although there are some dominant patterns in milestone sequence, there is also substantial diversity in trajectories. Although theoretical models proposing common linear sequences of stages in LGB+ identity development (e.g., Cass, 1979, 1984, 1996; Coleman, 1982) were noteworthy efforts during their time to advance scholarship in this understudied area, our findings and related scholarship underscore that LGB+ identity development is more diverse, dynamic, and complex than the stage models postulated (Eliason and Schope, 2007; Savin-Williams, 2011; Morgan, 2012; Savin-Williams and Cohen, 2015). Our results indicate that LGB+ identity development, like many aspects of human development, can be understood as a *cascade model* in which early sexual experiences serve as bases for and influence future sexual experiences in the life course (Diamond et al., 2015). For instance, attraction appears to be a foundational milestone that influences subsequent milestones of self-identification and sexual activity. Further, self-identifying as LGB+ is undoubtedly a necessary precursor of coming out to others as LGB+; one’s patterns of attraction and self-identification guide the romantic relationships individuals pursue; and one’s first coming out experience likely shapes the timing and form of subsequent disclosure events. There are multiple cascading patterns in sexuality development. Theory about the conditions or factors that shape particular sequences of milestones needs elaboration. Milestone sequences are undoubtedly influenced by biological, psychological, social, and cultural factors. Additional research could illuminate the combinations of variables that shape particular trajectories.

### Milestone Chronology by Sex, Race/Ethnicity, Sexual Orientation, and Birth Cohort

Findings indicated that, on average, males reached the milestones of attraction, self-identification, and sexual activity earlier than females; however, fewer differences were found in these milestones among Millennials. This finding may be explained by shifts in societal views about sexuality and/or females beginning puberty earlier than in past decades. From a sociocultural perspective, older birth cohorts (i.e., the Silent Generation, Baby Boomers, and Gen X) may have been highly influenced by gender role expectations. For men, a strong sex drive and pursuit of sexual encounters were valued in United States society as signs of robust masculinity, whereas women may have been influenced by messages that their sexuality should be minimized, sexually active young women were “sluts,” and sexual activity outside of monogamous committed relationships or marriage was unacceptable. National data show more sexually liberal and sex-positive views among Millennials than prior generations (Twenge et al., 2015). From a biological perspective, the average age that females begin puberty is earlier among Millennials than prior cohorts (Steingraber, 2007), which may explain the fewer differences by sex in initial milestones among Millennials. Generally, males and females came out and had their first romantic relationships at about the same ages, and females reached the main milestones in a shorter amount of time, compared to males. The more prolonged process for males could be due to males having higher levels of internalized stigma, which may delay coming out and pursuing a LGB+ relationship despite early experiences of queer attractions and encounters. Studies have found slightly higher levels of internalized stigma among gay and bisexual men (Mohr and Fassinger, 2000; Herek et al., 2009; Barnes and Meyer, 2012).

We found fewer differences in milestone timing between racial/ethnic groups. Nonetheless, Hispanic/Latinx people reached all of the main milestones first, except for initial sexual encounter, which Black/African American people experienced earliest. We also found that White people reached most of the milestones later than people of color, especially when compared with Black and Hispanic/Latinx people. These findings are consistent with evidence from the general population showing earlier sexual activity among Hispanic/Latinx and Black youth, as well as later sexual activity among Asian and White young people (O’Sullivan et al., 2007; Zimmer-Gembeck and Helfand, 2008). These differences may be due to differences in socialization about sexuality between racial/ethnic groups and the earlier onset of puberty for Black and Hispanic/Latinx youth (Ward and Wyatt, 1994; Sun et al., 2002; Meneses et al., 2006; Butts and Seifer, 2010).

Bisexual people generally reached most milestones slightly later compared to gay/lesbian people. This may be due to several factors. Given the sociocultural pressure of heteronormativity, bisexual peoples’ attractions to multiple genders, and their capacity to engage in sexual behaviors and relationships that may be viewed as heterosexual, there may be more denial, minimization, or uncertainty about their bisexual sexuality than for gay/lesbian people. In addition, because bisexuality as a legitimate sexual orientation has historically been questioned, with views that bisexuality is a transitional step between heterosexuality and homosexuality and that very few people are truly bisexual, people with an emerging bisexual identity may feel more confusion and self-doubt about their identity than people with monosexual orientations (Brown, 2002; Roberts et al., 2015; Monro et al., 2017). Longitudinal research indicates that bisexual people can experience more fluctuations in their attractions over time compared to gay/lesbian people (Diamond, 2008), which may also contribute to confusion and feeling uncertain about their bisexual identity. Bisexual people also face prejudice and discrimination from both the heterosexual community and gay/lesbian community, often related to invalidation, mistrust, and hypersexualization of a bisexual identity (Roberts et al., 2015). Such experiences likely contribute to a special form of internalized stigma (e.g., internalized binegativity/biphobia) that may also delay their sexual identity development.

Our results showed that the older the birth cohort, the later the milestones were reached. And, the older the cohort, the longer the duration between the milestones of attraction and romantic relationship. We also found that Millennials had a different milestone sequence than prior cohorts where Millennials self-identified as LGB+ following attraction, whereas other cohorts engaged in sexual activity before self-identifying. Further, Millennials engaged in sexual activity and came out at about the same age. Collectively, these findings demonstrate the influence of cohort and period effects on LGB+ identity development. Public opinion data show that Americans’ attitudes about homosexuality and LGB+ issues have improved over time (Saad, 2012; Hall and Rodgers, 2019; Pew Research Center, 2019a; Flores, 2020). Correspondingly, evidence shows that younger cohorts of Americans report more accepting attitudes about LGB+ people and issues, compared to older cohorts (Avery et al., 2007; Andersen and Fetner, 2008). More positive social climates undoubtedly facilitate individuals feeling comfortable exploring their sexuality, self-identifying as LGB+, and living openly as LGB+. Future research with Generation Z, the most recent cohort, may find that milestone timing is even earlier than Millennials, though with a similar sequence.

### Strengths and Limitations of the Review

This review used a rigorous approach to identify relevant studies by searching multiple databases using an expert-informed search string and screening over 3,200 records. Search records were independently screened by two screeners based on *a priori* inclusion criteria. The studies reviewed included formally published sources (e.g., peer-reviewed journal articles and book chapters) and several gray literature sources (i.e., unpublished dissertations and a think tank report) to minimize publication bias. Nonetheless, unpublished research may be underrepresented in this review. Although the bibliographic databases searched were large and directly relevant to the research questions, searching additional databases may have led to the discovery of other studies. In hindsight, additional search terms referring to some of the specific milestones (e.g., coming out) could have been used, which may have revealed additional pertinent studies. Meta-analysis results were based on the best available evidence among the studies; however, few studies possessed a high level of methodological rigor regarding internal and external validity. Therefore, caution should be taken when generalizing results because certain meta-analysis results are based on two rigorous studies and other results are based on several studies with notable limitations. Another complicating factor in the review was the inconsistent measurement of the milestones. By presenting the methodological characteristics of the studies in Supplementary Table 1 and the results in Supplementary Tables 2 and 3, readers are able to assess the methodological rigor and trustworthiness of the findings.

### Methodological Considerations of the Studies Reviewed

Systematic reviews not only summarize what is substantively known about a topic but also provide a critical appraisal of the state of the research on a topic. Based on our appraisal of the methodological characteristics of the studies, we identified several strengths among the studies. A substantial portion of the studies used large, national samples, and most studies had relatively large sample sizes. These sampling strengths are beneficial for the generalizability of the findings because large and national samples are more likely to be representative of the US population. The studies were generally balanced in terms of sex, with many having comparable numbers of males and females, as well as some studies that exclusively focused on men or women. Historically, LGB+ identity research has tended to focus on gay men, White gay men in particular, more so than other subgroups of the queer community (Eliason and Schope, 2007; Morgan, 2012). Studies also included sufficient representation of various birth cohorts aside from Generation Z, as well as participants of various ages.

Conversely, several prominent methodological limitations were identified among the studies. All of the studies measured milestones using retrospective, cross-sectional methods. Although such methods are highly feasible, they are not ideal for capturing events and experiences that unfold during development, particularly for respondents in middle or older adulthood who are recalling milestones that often occurred during adolescence or young adulthood. Recall error is a notable concern, especially for participants recalling events that occurred many years or even decades ago. Only a few studies used mixed methods to capture milestones and their contexts. Although quantitative methods are essential for capturing milestone chronology, qualitative methods can capture rich, in-depth data about the context and meaning of milestone experiences. Sampling issues and the representation of various subgroups within the LGB+ population were limited for some studies. Many samples were largely or primarily White, with limited representation of people of color. Numerous studies focused on gay/lesbian individuals, followed by bisexual individuals, with limited inclusion of other sexual minorities (e.g., queer and pansexual people). There was also limited measurement of important demographics, including gender identity, socioeconomic status, national origin, immigrant/citizenship status, ability/disability status, and religious background.

### Recommendations for Future Research

We anticipate that research on LGB+ identity development will continue to expand. In order to build upon and address gaps and limitations in the extant literature, we present six recommendations for future research. These recommendations are based on the critical appraisal of methodological characteristics and findings from the studies reviewed.

First, future research should improve sampling methods. Historically, LGB+ people have been a hard-to-reach, vulnerable, and hidden population for researchers (Meyer and Wilson, 2009; Ellard-Gray et al., 2015), although inclusion of this population has improved in recent decades. Nationally representative samples of LGB+ people that reflect the diversity of America are needed to produce generalizable knowledge. Online-, telephone-, and address-based sampling methods can facilitate samples that are national and more representative. Nonetheless, well-designed and well-executed non-probability community-based samples are still needed. Community-based studies can explore in-depth experiences from segments of the LGB+ community who are particularly hard-to-reach and/or have been underrepresented in extant research, including sexual minorities other than gays/lesbians (e.g., queer, bisexual, and pansexual people), transgender and nonbinary people, people of color, people living in rural areas, people living in the South and Mountain-Prairie region, and Generation Z. Community venue-based sampling, time-space sampling, respondent-driven sampling, adaptive sampling, and follow-back methods may be useful approaches in reaching these groups (Corliss et al., 2009a; Meyer and Wilson, 2009), as well as strategies to overcome barriers to participation (Elze, 2009; Rodríguez Rust, 2009; Ellard-Gray et al., 2015).

Second, more research is needed on intersectionality. The queer community is a heterogeneous population group and one’s sexual orientation identity intersects with many other identities and social locations (e.g., race/ethnicity, socioeconomic status, sex, gender identity, ability/disability status, immigrant/citizenship status, religious/spiritual orientation, and age group), which are associated with various social systems of privilege and oppression (e.g., racism, classism, sexism, cisgenderism, ableism, nativism, religism, and ageism). An intersectional approach can be incorporated in the conceptualization, sampling, data collection methods, and data analysis of a study (Bowleg, 2008; Warner, 2008; Cole, 2009).

Third, future research must use more rigorous study designs. Single or multiple cohort prospective longitudinal studies with appropriate time intervals to capture milestones would be the most rigorous designs, yet also the most resource-intensive. Cross-sequential studies are more feasible than longitudinal studies and more rigorous than cross-sectional studies. Another rigorous alternative that has been hardly used in LGB+ research is the life history/event calendar design. Life history/event calendar methods were developed to address concerns about recall error in traditional retrospective interviews, surveys, and questionnaires in the social/behavioral sciences (Freedman et al., 1988; Caspi et al., 1996; Glasner and van der Vaart, 2009). Rather than asking discrete questions about milestones *via* an interview or a self-administered questionnaire (e.g., “How old were you when you first identified as LGB?”), calendar methods use visual aids, cognitive recall strategies, and interactive data collection to minimize recall error (Belli, 1998; Axinn et al., 1999; Belli and Callegaro, 2009; Glasner and van der Vaart, 2009). Calendar methods can yield quantitative and qualitative retrospective life-course data and have several advantages over traditional retrospective methods (e.g., recall accuracy and data completeness) and over prospective longitudinal methods (e.g., no attrition and no testing effects). Though calendar methods provide advantages regarding feasibility and validity and are well-suited for addressing critical gaps in knowledge in this area, only a handful of studies have used calendar methods with LGB+ populations (Fisher, 2012, 2013; Fisher et al., 2014; Goldbach and Gibbs, 2015, 2017).

Fourth, regardless of the number and timing of data collection points, future studies should use more mixed-methods designs. Quantitative methods can capture important variables, including the timing, sequencing, durations between, and frequency of milestones. Despite the descriptive utility of mapping chronological signposts in the identity development process, exclusive use of quantitative methods misses important information about the context and meaning of milestones. Indeed, sexual orientation identity development involves diverse, complex, and dynamic developmental experiences and processes unfolding in psychological and social contexts over time. Qualitative methods can provide important data about the meaning of sexual orientation identities across individuals, groups, places, and times; ways individuals explore, process, and integrate their sexual orientation; and psychosocial contexts of milestones, such as individuals’ psychological reactions to milestones, ways social and cultural forces influence their experiences, changes in interpersonal relationships due to milestone experiences, ways individuals cope with difficult milestone experiences, and experiences that lead to adaptive psychosocial functioning and resilience.

Fifth, researchers should improve the measurement and reporting of milestones. Although dependent on the purpose of a study, researchers should endeavor to measure as many of the key milestones as possible to develop more comprehensive knowledge on LGB+ identity development trajectories. Measurement concerns about the construct validity of certain milestones, as discussed previously for attraction and sexual activity, can be addressed by using specific, multi-item measures. Given that LGB+ young people are disproportionate targets for sexual abuse and sexual assault (Saewyc et al., 2006; Friedman et al., 2011; Rothman et al., 2011), questions about sexual activity may need to distinguish between consensual and non-consensual encounters. Questions about coming out can ask about the very first time someone comes out to others as LGB+, as well as disclosure to specific people (e.g., parents, siblings, and friends) and social settings (e.g., school, workplace, neighborhood, and religious/spiritual community). Researchers could also allow respondents to indicate if they were outed (i.e., their identity was disclosed to someone else without their consent) or if their identity was inadvertently discovered by someone else. Although milestone research typically captures the first time a milestone occurs, a substantial minority of LGB+ people experience fluidity in their identity. Therefore, measures can be crafted so that they can capture milestones experienced more than once (e.g., someone initially identifies as lesbian, but later questions their identity and self-identifies as bisexual). In reporting the timing of milestones, researchers should continue to report means and standard deviations, but also medians and ranges. Medians are robust against outliers and skewed distributions, which occur in developmental data. Similarly, although standard deviations are useful statistics to understand the spread of the data, ranges in terms of minimum and maximum values convey the total span of the data.

Finally, although a number of researchers have investigated associations between milestone variables and psychosocial outcomes (e.g., Floyd and Stein, 2002; Friedman et al., 2008; Katz-Wise et al., 2017a; Rendina et al., 2019), more research is needed to examine how LGB+ identity development relates to mental, behavioral, and social outcomes. As described earlier in this paper, LGB+ people face disproportionately high rates of depression, anxiety, suicidal ideation and behavior, substance abuse, sexually transmitted infections, and disordered eating. Milestone variables may be directly and indirectly related to important psychosocial outcomes (e.g., mental health, substance use, life satisfaction, interpersonal wellbeing, relationship satisfaction, and self-esteem). In addition, key mediating and moderating variables need to be identified, which could inform the development of interventions. These mediating or moderating variables could include individual variables (e.g., age, race/ethnicity, gender, and religiosity), psychological variables (e.g., self-acceptance, internalized stigma, personal authenticity, identity centrality/prominence, and identity integration), interpersonal variables (e.g., outness, family reactions, social support, and involvement with the LGBTQ community), and sociocultural environmental variables (e.g., urbanicity/rurality, socio-political climate, and policy landscape). Such research should not rely on simplistic and reductionist analytic methods, but rather advanced methods, such as general structural equation modeling, latent profile analysis, latent curve modeling, regression modeling with interactions, machine learning for qualitative analysis, hierarchical linear modeling, and mixed methods. Understanding complex phenomena often requires complex methods.

### Conclusion

This systematic review and meta-analysis demonstrate the complexity of sexual orientation identity development for LGB+ people—an important dimension of human development involving many milestones that are cascading over time, shaped by biopsychosocial factors, and illustrative of common and unique developmental trajectories. Additional research is needed to further explicate these developmental journeys, which have significant implications for the wellbeing and lives of LGB+ people.

## Data Availability Statement

The raw data supporting the conclusions of this article will be made available by the authors, without undue reservation.

## Author Contributions

WH conceptualized the study and led the study design. WH, HD, and NP analyzed the data, interpreted the results, and wrote and edited parts of the manuscript. All authors approved the manuscript as submitted.

## Funding

Research reported in this article was supported by the National Institute of Minority Health and Health Disparities of the National Institutes of Health under award number R01MD015109-01A1.

## Conflict of Interest

The authors declare that the research was conducted in the absence of any commercial or financial relationships that could be construed as a potential conflict of interest.

## Publisher’s Note

All claims expressed in this article are solely those of the authors and do not necessarily represent those of their affiliated organizations, or those of the publisher, the editors and the reviewers. Any product that may be evaluated in this article, or claim that may be made by its manufacturer, is not guaranteed or endorsed by the publisher.
