# Supplementary material for: Sexual Orientation Identity Development Milestones Among Lesbian, Gay, Bisexual, and Queer People: A Systematic Review and Meta-Analysis
Source: Front Psychol. 2021 Oct 21;12:753954. doi: 10.3389/fpsyg.2021.753954 (PMC8581765; doi:10.3389/fpsyg.2021.753954)
Supplement: Supplementary file 1 [file Table_1.docx]

Supplementary Material

Table 1

*Summary of Studies Included in the Systematic Review*

| **Study Citation(s)** | **Study Design and Sampling** | **Sample Size** | **Sample Age** | **Sample Demographics** | **Year(s) of Data Collection** | **Birth Cohort(s) in Sample** |
| --- | --- | --- | --- | --- | --- | --- |
| Calzo et al., 2011 | Quantitative study of two cross-sectional samples using population-based sampling in California | 1,260 | *M* = 49.3 (*SD* = 12.3), range: 18-84 | 53% male, 47% female; 73% gay/lesbian, 27% bisexual; 83% White, 8% Latinx, 5% Black, 3% Asian, 2% American Indian | 2003 and 2007 | Silent, Boomers, and Gen X |
| Corliss et al., 2009b | Quantitative cross-sectional study using convenience sampling in the Los Angeles and San Francisco Bay areas | 2,001 | *M* = 40  (*SD* = 12), range: 18-72 | 100% female; 83% gay/lesbian, 11% bisexual, 6% other; 67% White, 13% Latinx, 10% Black, 7% Asian, 3% other | 2004-2005 | Silent, Boomers, Gen X, and Millennials |
| D'Augelli, 2002, 2003, 2006; D'Augelli et al., 1998; D'Augelli & Hershberger, 1993 | Quantitative study of two cross-sectional samples using convenience sampling in the United States and Canada | 542 | *M* = 19.1 (*SD* = 1.5), range: 14-21 | 62% male, 38% female; 74% gay/lesbian, 26% bisexual; 75% White, 8% Black, 4% Latinx, 1% American Indian, 12% other | 1987-1989 and 1995-1997 | Gen X and Millennials |
| D'Augelli et al., 2008; Grossman et al., 2014 | Quantitative study using the baseline wave of a longitudinal study using convenience sampling in the New York City area | 528 | *M* = 17 (*SD* = 1.3),  range: 15-19 | 52% male, 48% female; 53% bisexual, 47% gay/lesbian; 45% Latinx, 62% White, 25% Black, 10% multiracial, 3% Asian | 1999-2001 | Millennials |
| Diamond, 1998, 2003, 2005 | Mixed-methods study using the baseline wave of a longitudinal study using purposive sampling in New York state | 89 | *M* = 20 (*SD* = 2.0), range: 16-23 | 100% female; 43% lesbian, 30% bisexual, 27% other; 16% people of color, 84% White | 1995 | Gen X |
| Dirkes et al., 2016 | Quantitative study of two cross-sectional samples using convenience and respondent-driven sampling in the Chicago area | 820 | *M* = 35.6 (*SD* = 11.9),  range: 18-83 | 100% female; 60% lesbian, 20% mostly lesbian, 16% bisexual, 4% other; 37% White, 35% Black, 25% Latinx, 4% other | 2000-2001 and 2010-2012 | Silent, Boomers, Gen X, and Millennials |
| Drasin et al., 2008 | Quantitative cross-sectional study using convenience sampling in the United States | 2,402 | *M* = 38.3  (*SD* = 11.1),   \| range: 18-83 \| \| --- \| | 100% male; 100% gay; 90% White, 6% Latinx, 2% Black, 1% Asian, 1% other | 1994 | Silent, Boomers, and Gen X |
| Dubé, 1997, 2000; Dubé & Savin-Williams, 1999 | Quantitative cross-sectional study using convenience and snowball sampling in New York state | 139 | *M* = 21.3 (*SD* = 2.6),  range: 16-26 | 100% male; 100% gay or bisexual; 40% White, 19% Black, 24% Latinx, 17% Asian | 1996 | Gen X |
| Dunlap, 2016 | Quantitative cross-sectional study using convenience sampling in the United States | 1,131 | *M* = 39.3 (*SD* = 15.7),  range: 18-85 | 58% female, 42% male; 73% gay/lesbian, 11% queer, 8% bisexual, 1% pansexual, 7% other; 76% White, 8% Latinx, 4% Black, 4% multiracial, 2% Asian, 2% American Indian | 2010-2011 | Silent, Boomers, Gen X, Millennials, and Gen Z |
| Fisher, 2012 | Quantitative cross-sectional study using convenience sampling in a Midwest city | 16 | *M* = 18.4 (*SD* = 1.6),  range: 15-20 | 63% male, 19% female, 19% transgender; 75% gay/lesbian, 12.5% queer, 12.5% unsure; 50% Black, 25% White, 12.5% multiracial, 12.5% Latinx | 2006 | Millennials |
| Floyd & Bakeman, 2006 | Quantitative cross-sectional study using convenience sampling in Atlanta, Georgia | 767 | *M* = 34.7  (*SD* = 9.5),  range: 18-74 | 46% female, 54% male; 93% gay/lesbian, 7% bisexual; 76% White, 11% Black, 13% other | 2001 | Boomers, Gen X, and Millennials |
| Floyd & Stein, 2002 | Quantitative cross-sectional study using convenience sampling in the Midwest and Southeast | 72 | *M* = 20.9 (*SD* = 2.9), range: 16-27 | 50% male, 50% female; 76% gay/lesbian, 24% bisexual; 79% White, 7% Asian, 6% Black, 3% American Indian, 6% other | 1994 | Gen X |
| Fox, 1993 | Quantitative cross-sectional study using convenience sampling in the United States | 835 | *M* = 30.3 (*SD* = 8.7),  range: 16-59 | 58% female, 42% male; 100% bisexual; 90% White, 2% Black, 2% Latinx, 1% American Indian, 4% multiracial | 1990-1991 | Silent, Boomers, and Gen X |
| Fredriksen-Goldsen et al., 2017 | Quantitative study using the baseline wave of a longitudinal study using purposive sampling in the United States | 2,450 | *M* = 61.5  (*SD* = 12.1), range: 50-98 | 43% female, 57% male; 70% gay/lesbian, 17% bisexual, 13% other; 78% White, 9% Black, 9% Latinx, 4% other | 2014 | Silent and Boomers |
| Friedman et al., 2008; Friedman et al., 2006 | Quantitative cross-sectional study using disproportionate stratified and adaptive sampling in Los Angeles, Chicago, San Francisco, and New York City | 1,383 | *M* = 32.5 (*SD* = 5.04), range: 18-40 | 100% gay/bisexual men; 77% White, 11% Latinx, 6% Asian, 4% Black, 3% American Indian | 1996-1998 | Boomers and Gen X |
| Grov et al., 2006 | Quantitative cross-sectional study using convenience sampling in New York City and Los Angeles | 2,733 | *M* = 37.4 (*SD* = 11.0), range: 18-84 | 15% female, 85% male; 100% gay/lesbian or bisexual; 10% Black, 6% Asian, 15% Latinx, 62% White, 7% other | 2003-2004 | Silent, Boomers, and Gen X |
| Grov et al., 2018 | Quantitative study using Wave 2 of a longitudinal study using convenience sampling in the United States | 1,023 | *M* = 40.2 (*SD* NR), range: 19-80 | 100% male; 95% gay, 5% bisexual; 71% White, 8% Black, 13% Latinx, 5% Asian, 1% American Indian, 3% other | 2015 | Silent, Boomers, Gen X, and Millennials |
| Herdt & Boxer 1993 | Mixed-methods cross-sectional study using convenience sampling in Chicago | 202 | *M* and *SD* NR; range: 14-20 | 70% male, 30% female; 100% gay/lesbian; 30% Black, 40% White, 13% Latinx, 3% Asian, 14% multiracial | 1987-1988 | Gen X |
| Herek et al., 2010 | Quantitative cross-sectional study using probability sampling in the United States | 662 | *M* = 39 (*SD* NR), range: 18-89 | 47% female, 53% male; 49% gay/lesbian, 51% bisexual; 65% White, 16% Black, 13% Latinx, 7% other | 2005 | Silent, Boomers, Gen X, and Millennials |
| Hoenig, 2016 | Quantitative study using the baseline wave of a longitudinal study using convenience sampling in three large U.S. cities | 835 | *M* = 18.3 (*SD* = 1.8), range: 15-21 | 47% male, 53% female; 54% gay/lesbian, 46% bisexual; 5% Asian, 25% Black, 22% Latinx, 20% White, 24% multiracial, 5% other | 2012-2013 | Millennials |
| Katz-Wise et al., 2017a; Katz-Wise et al., 2017b | Quantitative study using one wave of a longitudinal study using convenience sampling in the United States | 1,461 | *M* = 25.4 (*SD* = 1.6), range: 22-30 | 75% female, 25% male; 75% mostly heterosexual, 11% bisexual, 14% gay/lesbian; 91% White, 9% people of color | 2010 | Millennials |
| Maguen et al., 2002 | Quantitative cross-sectional study using convenience sampling in the Southeast | 117 | *M* = 20 (*SD* = 2.8), range: 14-27 | 54% male, 46% female; 75% gay/lesbian, 22% bisexual, 3% queer; 75% White, 11% Black, 5% Latinx, 4% multiracial, 3% Asian, 1% other | 1997 | Gen X and Millennials |
| Martos et al., 2015 | Quantitative study using baseline wave of a longitudinal study using quota and snowball sampling in New York City | 396 | *M* = 32.4 (*SD* = 9.2), range: 18-59 | 50% men, 50% women; 82% gay/lesbian, 18% bisexual; 34% White, 33% Black, 33% Latinx | 2004-2005 | Boomers, Gen X, and Millennials |
| Morris et al., 2002 | Quantitative cross-sectional study using convenience sampling in the United States | 2,431 | *M* = 36.3 (*SD* = 5.1), range: 15-83 | 100% female; 55% bisexual, 44% lesbian/gay; 76% White, 9% Black, 7% Latinx, 3% American Indian, 3% Asian, 2% other | 1994-1995 | Silent, Boomers, and Gen X |
| Parks & Hughes, 2007; Parks et al., 2004 | Quantitative study using baseline wave of a longitudinal study using convenience sampling in Chicago | 426 | *M* = 38.4 (*SD* = 11.6), range: 18-83 | 100% female; 100% gay/lesbian; 47% White, 28% Black, 20% Latinx, 5% other | 2000-2001 | Silent, Boomers, and Gen X |
| Pew Research Center, 2013a | Quantitative cross-sectional study using probability sampling in the United States | 1,197 | *M* and *SD* NR; 30% ages 18-29, 39% ages 30-49, 23% ages 50-64, 9% ages 65 or older | 50% male, 49% female; 56% gay/lesbian; 44% bisexual; 66% White, 10% Black, 17% Latinx, 7% other | 2013 | Boomers, Gen X, and Millennials |
| Rendina et al., 2019 | Quantitative study using baseline wave of a longitudinal study using convenience sampling in the New York City area | 374 | *M* = 36.9 (*SD* = 11.4), range: 18-73 | 100% male; 88% gay, 12% bisexual; 51% White, 20% Black, 14% Latinx, 16% multiracial or other | 2011 | Boomers, Gen X, and Millennials |
| Rosario et al., 1996; Schrimshaw et al., 2006 | Quantitative study using baseline wave of a longitudinal study using convenience sampling in New York City | 156 | *M* = 18.3 (*SD* = 1.7), range: 14-21 | 51% male, 49% female; 69% gay/lesbian, 28% bisexual, 3% other; 35% Black, 37% Latinx, 5% Asian, 22% White, 2% other | 1993-1994 | Gen X |
| Savin-Williams, 1995, 1998; Savin-Williams & Diamond, 2000^a^ | Quantitative study of three cross-sectional samples using convenience sampling in New York state and the Detroit area | 180 | *M* = 20.4 (*SD* = 2.4), range: 14-25 | 100% male; 62% gay, 33% mostly gay, 4% bisexual, 1% mostly heterosexual; 77% White, 10% Black, 7% Latinx, 4% Asian, 2% American Indian, 1% other | 1988-1991 and 1994 | Gen X |
| Savin-Williams & Diamond, 2000^b^ | Quantitative cross-sectional study using convenience sampling in New York state | 78 | *M* = 20.8 (*SD* = 1.7), range: 17-25 | 100% female; 35% gay/lesbian, 33% bisexual, 13% questioning, 19% other; 78% White, 3% Black, 13% Asian, 3% Latinx, 4% multiracial or other | 1997-1998 | Gen X |

*Note*. NR = not reported. Silent = Silent Generation. Boomers = Baby Boomer Generation. Gen X = Generation X. Millennials = Millennial Generation or Generation Y. Gen Z = Generation Z. ^a^ Male sample from this study (Savin-Williams & Diamond, 2000) was included in Savin-Williams (1998). ^b^ Female sample only.

Table 2

*Summary of Studies on the Timing of LGB+ Identity Development Milestones and Meta-Analysis Mean Effect Sizes*

| **Study Citation(s)** | **Milestone Mean Age (*SD*)** | | | | | | | |
| --- | --- | --- | --- | --- | --- | --- | --- | --- |
|  | Attraction | Questioned Orientation | Self-Identify | Sexual Activity | Came Out to Others | Came Out to Parents | Came Out to Other Family Members | Romantic Relationship |
| Calzo et al., 2011 | 14.9 (7.5) |  | 19.7 (7.7) | 20.6 (7.6) | 23.9 (8.3) |  |  |  |
| Corliss et al., 2009b | 16.0 (8.0) |  |  | 21.0 (7.0) | 23.0 (8.0) |  |  |  |
| D'Augelli, 2002, 2003, 2006; D'Augelli et al., 1998; D'Augelli & Hershberger, 1993 | 10.4 (3.8) |  | 15.2 (2.8) | 14.9 (3.7) | 16.7 (2.0) | 17.3 (2.1) | 17.3 (2.2) | 17.3 (2.0) |
| D'Augelli et al., 2008; Grossman et al., 2014 | 12.4 (2.9) |  | 14.0 (2.3) |  | 14.6 (2.0) | 14.9 (2.2) |  |  |
| Diamond, 1998, 2003, 2005 | 15.0 (3.5) | 16.0 (2.6) | 17.6 (2.1) | 18.0 (2.1) |  |  |  |  |
| Dirkes et al., 2016 |  | 15.2 (6.8) | 20.8 (7.8) |  | 22.0 (8.2) |  |  |  |
| Drasin et al., 2008 | 11.6 (NR) |  | 16.9 (NR) | 15.5 (NR) | 22.5 (NR) |  |  |  |
| Dubé, 1997, 2000; Dubé & Savin-Williams, 1999 | 10.0 (4.3) |  | 15.8 (3.5) | 15.4 (4.2) | 17.0 (2.4) |  |  | 18.0 (2.5) |
| Dunlap, 2016 | 13.1 (6.0) |  | 18.1 (6.9) | 18.9 (6.7) | 20.7 (6.4) |  | 23.68 (7.3) | 22.5 (7.2) |
| Fisher, 2012 | 10.6 (3.7) |  | 12.6 (4.8) | 13.4 (4.1) | 15.3 (1.9) |  |  |  |
| Floyd & Bakeman, 2006 |  | 13.2 (6.2) | 19.7 (6.8) | 19.8 (6.4) | 22.8 (6.8) | 22.4 (6.7) |  |  |
| Floyd & Stein, 2002 | 10.4 (3.4) |  | 16.1 (3.9) | 16.3 (4.5) | 17.3 (2.5) | 18.1 (2.7) | 18.7 (2.5) | 18.5 (2.7) |
| Fox, 1993 | 14.9 (6.7) | 17.7 (7.0) | 22.4 (7.9) | 18.5 (7.5) | 22.6 (7.2) | 25.0 (7.2) | 26.0 (7.5) | 22.9 (7.5) |
| Fredriksen-Goldsen et al., 2017 |  |  | 20.8 (NR) |  | 27.5 (NR) |  |  |  |
| Friedman et al., 2008; Friedman et al., 2006 | 10.4 (NR) | 13.9 (NR) | 18.6 (NR) | 16.6 (NR) | 20.8 (NR) |  |  |  |
| Grov et al., 2006 |  |  | 17.8 (3.2) | 18.2 (3.7) | 20.7 (3.5) |  |  |  |
| Grov et al., 2018 | 11.5 (NR) |  | 16.6 (NR) | 17.6 (NR) | 19.0 (NR) |  |  |  |
| Herdt & Boxer, 1993 | 9.7 (3.6) |  | 16.0 (2.3) | 13.6 (4.1) |  |  |  |  |
| Herek et al., 2010 |  |  | 17.3 (NR) |  | 20.9 (NR) |  |  |  |
| Hoenig, 2016 | 12.3 (3.7) |  | 14.6 (2.6) | 15.1 (2.9) | 14.4 (2.4) |  |  |  |
| Katz-Wise et al., 2017a, 2017b | 16.3 (4.4) |  | 17.4 (4.0) | 17.8 (2.8) |  |  |  |  |
| Maguen et al., 2002 | 10.9 (4.5) |  |  | 15.6 (4.2) | 16.6 (2.6) |  |  |  |
| Martos et al., 2015 | 11.3 (4.8) |  | 16.2 (6.2) |  | 19.7 (5.2) | 21.2 (6.1) | 21.2 (6.1) | 18.4 (6.4) |
| Morris et al., 2002 |  | 17.7 (4.7) | 22.5 (6.4) | 21.8 (4.9) | 23.7 (5.9) | 25.5 (3.6) | 26.2 (4.1) |  |
| Parks & Hughes, 2007; Parks et al., 2004 |  | 15.9 (7.3) | 22.3 (8.1) |  | 23.8 (8.5) |  |  | 25.5 (7.7) |
| Pew Research Center, 2013a |  | 13.2 (7.1) | 18.1 (8.0) |  | 21.7 (8.5) |  |  |  |
| Rendina et al., 2019 | 10.0 (NR) |  | 15.3 (NR) | 17.7 (NR) |  |  |  |  |
| Rosario et al., 1996; Schrimshaw et al., 2006 | 10.7 (3.6) | 13.3 (3.5) | 15.0 (2.6) | 13.6 (3.8) |  |  |  |  |
| Savin-Williams, 1995, 1998; Savin-Williams & Diamond, 2000 | 8.0 (3.3) |  | 16.9 (3.0) | 14.1 (4.1) | 17.9 (2.5) |  | 18.7 (2.5) | 18.3 (2.7) |
| Savin-Williams & Diamond, 2000 | 9.0 (4.1) |  | 17.6 (2.1) | 16.4 (4.4) | 17.9 (1.9) |  |  |  |
|  | **Meta-Analysis Results by Milestone** | | | | | | | |
|  | Attraction | Questioned Orientation | Self-Identify | Sexual Activity | Came-Out to Others | Came-Out to Parents | Came-Out to Other Family Members | Romantic Relationship |
| **Mean Effect Size** [95% Confidence Interval] | **12.66** [10.05, 15.26] ^a^ | **13.22** [12.80, 13.63] ^b^ | **17.79** [11.62, 23.95] ^c^ | **18.06** [17.55, 18.57] ^a^ | **19.59** [17.17, 22.00] ^c^ | **22.60** [11.18, 34.02] ^a^ | **23.29** [16.68, 29.91] ^a^ | **20.88** [13.15, 28.61] ^a^ |

*Note*. A blank cell indicates that a milestone was not measured in a study. NR = not reported.

^a^ The meta-analysis results are based on studies from the second tier of methodological rigor (D’Augelli, 2002; Drasin et al., 2008; Dunlap, 2016; Fox, 1993; Fredriksen-Goldsen et al., 2017; Grov et al., 2018; Katz-Wise et al., 2017a; Morris et al., 2002).

^b^ A meta-analysis was not conducted for this milestone because just one of the two studies representing the best evidence measured this milestone; thus, the effect size and confidence interval presented is from that study (Pew Research Center, 2013a).

^c^ The meta-analysis results are based on studies representing the best available evidence (Herek et al., 2010; Pew Research Center, 2013a).

Table 3

*Meta-Analysis Results of Milestone Ages by Sex, Race/Ethnicity, Sexual Orientation, and Birth Cohort*

| Comparison Characteristic | **Mean Effect for each Milestone [95% Confidence Interval]** | | | | |
| --- | --- | --- | --- | --- | --- |
|  | Attraction | Self-Identify | Sexual Activity | Came Out to Others | Romantic Relationship |
| *Sex* ^a^ |  |  |  |  |  |
| Female | **15.26** [12.33, 18.19] | **19.11** [15.73, 22.50] | **19.93** [15.49, 24.37] | **21.85** [20.98, 22.71] | **20.64** [11.59, 29.69] |
| Male | **12.86** [9.88, 15.83] | **16.09** [15.15, 17.04] | **16.83** [16.30, 17.37] | **22.25** [19.87, 24.62] | **21.35** [13.26, 29.44] |
| *Race/Ethnicity* ^b^ |  |  |  |  |  |
| Asian American | **11.27** [6.36, 16.17] | **17.07** [13.41, 20.73] | **16.75** [5.33, 28.16] | **20.34** [12.49, 28.20] | **21.14** [12.06, 30.23] |
| Black/African American | **12.27** [11.10, 13.44] | **17.72** [14.73, 20.71] | **14.99** [7.95, 22.03] | **19.53** [13.35, 25.70] | **20.45** [9.76, 31.14] |
| Hispanic/Latinx | **10.55** [6.18, 14.93] | **16.70** [12.79, 20.61] | **15.14** [12.65, 17.63] | **19.12** [11.28, 26.95] | **19.79** [10.44, 29.13] |
| White | **11.42** [8.84, 14.00] | **18.35** [13.61, 23.09] | **15.34** [12.89, 17.80] | **21.18** [10.60, 31.75] | **21.18** [9.57, 32.79] |
| *Sexual Orientation* ^c^ |  |  |  |  |  |
| Bisexual | **16.87** [12.23, 21.50] | **19.35** [16.12, 22.58] | **17.63** [16.65, 18.61] | **21.55** [19.88, 23.23] | Not enough data |
| Gay/Lesbian | **15.14** [10.53, 19.74] | **17.19** [11.88, 22.49] | **16.71** [15.05, 18.38] | **21.28** [14.92, 27.65] | Not enough data |
| *Birth Cohort* ^d^ |  |  |  |  |  |
| Silent Generation | **15.04** [6.31, 23.78] | **21.76** [19.55, 23.96] | **21.94** [15.35, 28.54] | **28.38** [23.40, 33.36] | **29.23** [19.84, 39.34] |
| Baby Boomers | **13.05** [8.16, 17.93] | **20.08** [18.09, 22.07] | **19.06** [15.69, 22.43] | **23.69** [21.11, 26.28] | **24.27** [11.53, 37.01] |
| Generation X | **12.65** [9.01, 16.29] | **18.03** [16.15, 19.91] | **17.48** [12.71, 22.24] | **19.47** [15.37, 23.58] | **20.07** [16.91, 21.27] |
| Millennials | **11.97** [6.06, 17.87] | **15.36** [13.44, 17.29] | **16.90** [11.82, 21.98] | **17.24** [15.65, 18.82] | **17.86** [16.98, 20.60] |

^a^ The meta-analysis results for females and males are based on data from these studies: D’Augelli, 2002; Dunlap, 2016; Fox, 1993; Fredriksen-Goldsen et al., 2017; Herek et al., 2010; Katz-Wise et al., 2017a.

^b^ The meta-analysis results for racial/ethnic groups are based on data from these studies: Dubé & Savin-Williams, 1999; Fredricksen-Goldsen et al., 2017; Hoenig, 2016; Martos et al., 2015; Parks et al., 2004.

^c^ The meta-analysis results for sexual orientation identities are based on data from these studies: Fredriksen-Goldsen et al., 2017; Herek et al., 2010; Katz-Wise et al., 2017a; Pew Research Center, 2013a.

^d^ The meta-analysis results for birth cohorts are based on data from these studies: Dunlap, 2016; Fox, 1993; Fredrickson-Goldsen et al., 2017; Grov et al., 2018; Pew Research Center, 2013a.
